# Clinical Application of Cell-Based Approaches in Maxillary Sinus Floor Augmentation: A Systematic Review and Meta-Analysis

**DOI:** 10.3390/bioengineering12111209

**Published:** 2025-11-05

**Authors:** Sung-Hoon Han, Saet-Byeol Han, Greg Shinho Park, Na Jin Kim, Won-Jong Park, Jun-Beom Park

**Affiliations:** 1 Department of Orthodontics, Seoul St. Mary’s Hospital, College of Medicine, The Catholic University of Korea, Seoul 06591, Republic of Korea; scherazade@hanmail.net; 2 Graduate School of Clinical Dental Science, The Catholic University of Korea, Seoul 06591, Republic of Korea; 13star_z@naver.com; 3 Orthodontics, Graduate School of Clinical Dental Science, The Catholic University of Korea, Seoul 06591, Republic of Korea; gpark154@gmail.com; 4 Medical Library, College of Medicine, The Catholic University of Korea, Seoul 06591, Republic of Korea; kimnj@catholic.ac.kr; 5 Department of Oral and Maxillofacial Surgery, Seoul St. Mary’s Hospital, College of Medicine, The Catholic University of Korea, Seoul 06591, Republic of Korea; 6 Department of Periodontics, College of Medicine, The Catholic University of Korea, Seoul 06591, Republic of Korea; 7 Dental Implantology, Graduate School of Clinical Dental Science, The Catholic University of Korea, Seoul 06591, Republic of Korea; 8 Department of Medicine, Graduate School, The Catholic University of Korea, Seoul 06591, Republic of Korea

**Keywords:** sinus floor augmentation, stem cells, dental implants

## Abstract

Maxillary sinus floor augmentation is frequently performed to increase bone height for dental implants, with stem cells suggested to boost bone regeneration. Consequently, this study aimed to assess the effects of incorporating stem cells in maxillary sinus floor augmentation. Two reviewers conducted an extensive search using a mix of controlled vocabulary (MeSH) and free-text terms to locate published systematic reviews. Searches were conducted in three major electronic databases (Medline via PubMed, Embase, and Cochrane database) up to July 2025. Initially, 250 articles were found, but only five studies met inclusion criteria for meta-analysis. The meta-analysis revealed a pooled standardized mean difference in new bone formation of 1.06 (95% confidence interval of −0.31 to 2.44). In a subgroup analysis comparing mesenchymal stem cells with autogenous bone, the pooled standardized mean difference was 0.88 (95% confidence interval of 0.34 to 1.42). The study’s results indicated a positive trend towards better outcomes with the use of mesenchymal stem cells, although the effect was not statistically significant at the pooled level. Additionally, combining stem cells with xenograft may yield more favorable results compared to using autogenous bone with xenograft. These findings suggest potential clinical advantages, highlighting the need for further standardized research to verify long-term outcomes.

## 1. Introduction

Maxillary sinus floor augmentation, often referred to as a sinus lift or sinus floor elevation, is a surgical technique aimed at increasing bone height in the upper jaw’s posterior area to facilitate dental implant placement [1]. Although bone grafting materials are typically employed in sinus augmentation, incorporating stem cells into this process is a subject of ongoing research and development [2]. In a histomorphometric study comparing new bone formation following maxillary sinus lift using both lateral and crestal approaches with periosteal mesenchymal stem cells (MSCs) and beta-tricalcium phosphate, patients who received grafts with MSCs exhibited over 60% more bone formation than those who received grafts without stem cells [3]. A recent investigation found that MSCs seeded onto poly(ε-caprolactone)-β-tricalcium phosphate scaffolds, combined with platelet-rich plasma, led to a significantly higher bone–implant contact ratio compared to scaffolds without MSCs [4]. However, another recent study using allogeneic adipose tissue-derived stem cells indicated that adding stem cells to deproteinized bovine bone mineral did not enhance new bone formation or bone-to-implant contact compared to bone mineral alone in sinus floor augmentation procedures [5]. A previous review revealed no significant differences in implant survival rates or bone regeneration efficacy between stem cell-based grafts and traditional grafts, although stem cells might encourage greater bone formation six months after the procedure [6].

The inclusion of stem cells in sinus floor augmentation procedures holds promise for improving bone regeneration to support dental implant placement; however, the evidence is mixed, and further research is necessary to confirm their effectiveness and safety. Consequently, this study was conducted to systematically evaluate the clinical effects of cell-based approaches in maxillary sinus floor augmentation and to determine whether the incorporation of stem or progenitor cells enhances bone regeneration compared with conventional approaches. Additionally, the study sought to assess the quality of existing evidence, analyze the risk of bias and identify limitations that may influence the clinical translation of cell-based regenerative strategies.

## 2. Materials and Methods

### 2.1. Protocol and Eligibility Criteria

This systematic review adheres to the guidelines outlined in the Preferred Reporting Items for Systematic Reviews and Meta-Analyses (PRISMA) statement, as referenced in [7]. Our systematic review was conducted with careful adherence to the research methodologies of the PRISMA Statement. We gathered comprehensive data through thorough analysis and transparent reporting, leading to an extensive and unbiased assessment of the topic under study. The study protocol was registered in advance with PROSPERO (registration number: CRD42024589914).

Question: Does the additional use of stem cells make a difference in maxillary sinus augmentation?

Participants: Patients who underwent sinus floor augmentation.

Interventions: The application of graft material combined with stem cells.

Comparisons: The application of graft material without stem cells.

Outcomes: The increase in bone height, the percentage of new bone formation, the implant survival rate, and the marginal bone loss following implant placement after 3, 6, and 12 months.

Study design: Randomized controlled trials, restricted to studies involving adults.

The criteria for inclusion and exclusion are detailed below.

Inclusion Criteria:

- Studies involving sinus augmentation and stem cells.
- Cases with dental implantation.
- Studies focused on maxillary posterior area.

Exclusion Criteria:

- Studies on sinus augmentation that do not involve the use of stem cells.
- Patients who have not undergone dental implant procedures.
- Patients with maxillary dentures.
- Studies that did not provide the necessary data as mean and standard deviation for conducting a meta-analysis.

### 2.2. Information Sources and Search Strategy

An expert reviewer, NJK, associated with a library, initiated a preliminary search using a mix of controlled vocabulary (MeSH) and free-text terms to locate pertinent published studies. This search spanned three primary electronic databases: Medline via PubMed, the Cochrane Database, and Embase, with the search extending up to July 2025. Following this, two additional reviewers, HSH and WJP, executed a thorough search employing the same method—integrating controlled vocabulary (MeSH) and free-text terms to find published materials. A manual search was also performed to examine the references of all retrieved full-text articles, ensuring no relevant studies were overlooked during the electronic search. The search results were then transferred to EndNote reference management software (Version 21, Clarivate, Philadelphia, PA, USA) for the removal of duplicates. The search strategy was tailored to fit the specific needs of each database, with further details available in Supplementary Material Table S1.

### 2.3. Study Selection and Data Extraction

The titles and abstracts of the retrieved papers were independently screened by two reviewers (HSH and WJP) against the eligibility criteria in a blinded fashion. Subsequently, the full texts of the remaining articles were reviewed independently and in duplicate by the same two reviewers before final inclusion. Data extraction was conducted independently from the included studies based on the PICOS framework and organized into the following categories: general information (author name, publication year), sites (number of treatment), intervention/comparison (stem cell type, type of scaffold), and outcomes (new bone formation) at post-treatment.

### 2.4. Risk of Bias Assessment

The reviewers evaluated the included randomized studies using the Cochrane Risk-of-Bias (ROB 2.0) tool. The checklist addressed key areas, including the randomization process (selection bias), deviations from intended interventions (performance bias), missing outcome data (attrition bias), outcome measurement (detection bias), selection of reported results (reporting bias), and overall bias. Each study’s risk of bias was categorized as low risk, some concerns, or high risk. The quality of the eligible studies was independently assessed by two reviewers (HSH and WJP).

### 2.5. Data Synthesis and Analysis

The meta-analysis was performed using R (Version 4.3.2; R Project for Statistical Computing), with the standardized mean difference (SMD) and 95% confidence interval (CI) as summary statistics. A random-effects model was applied for the analysis, with the level of significance set at 0.05. Heterogeneity across studies was assessed using the *I*^2^ statistic and the chi-square test.

### 2.6. Assessment of Certainty of Evidence

The certainty of evidence for the primary outcomes was evaluated following the GRADE (Grading of Recommendations, Assessment, Development, and Evaluation) framework. This approach offers a systematic method to appraise the overall quality of evidence by considering multiple domains, including study design, risk of bias, inconsistency, indirectness, imprecision, and potential publication bias. In this study, the initial certainty for each outcome was rated as “high,” given that all included trials were randomized controlled studies. Downgrading was applied when specific concerns were identified according to predefined criteria within each domain.

## 3. Results

### 3.1. Study Selection and Data Extraction

**Figure 1:**
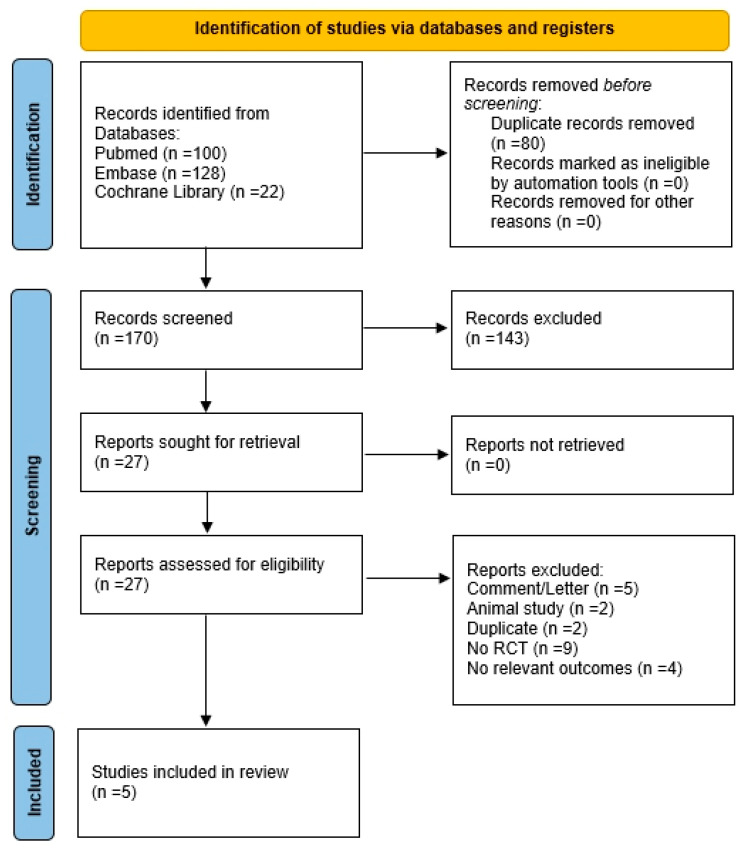
Flowchart illustrating the article selection process for the systematic review. This chart illustrates the identification, screening, and inclusion/exclusion of studies according to the predefined criteria.

A total of 250 records were initially located through database searches (PubMed, n = 100; Embase, n = 128; Cochrane database, n = 22). After removal of 80 duplicates, 170 records were screened, and 143 were excluded. Twenty-seven reports were sought for retrieval, all of which were successfully retrieved. Following eligibility assessment, 22 reports were excluded (comment/letter, n = 5; animal studies, n = 2; duplicate, n = 2; not randomized controlled trials, n = 9; no relevant outcomes, n = 4). Ultimately, five studies were included for the analysis. Figure 1 presents a flowchart of the study selection process, and Supplementary Material Table S2 lists the excluded articles along with the reasons for their exclusion. The main characteristics of the included studies are shown in Table 1.

### 3.2. Risk of Bias Assessment

**Figure 2:**
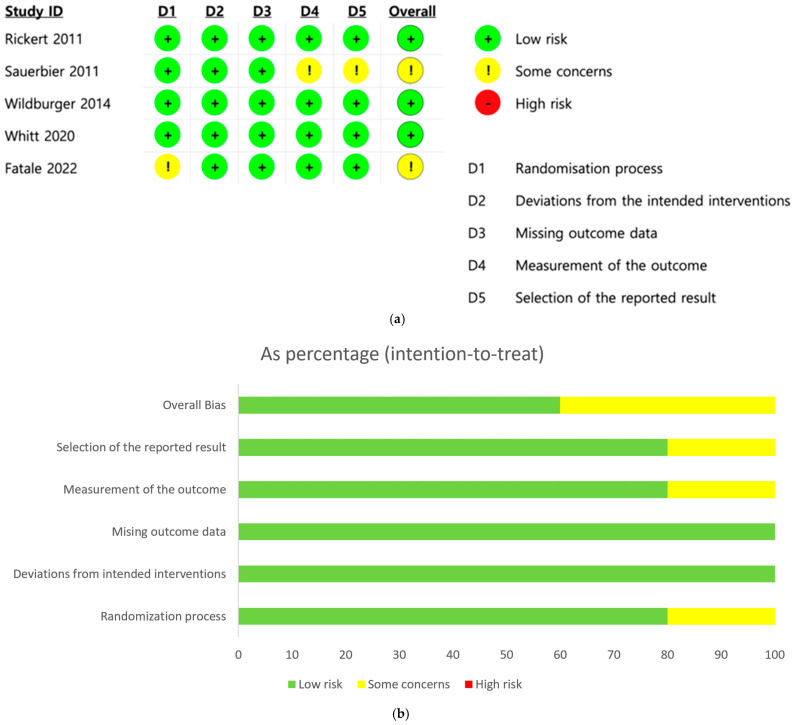
Evaluation of risk of bias. (**a**) Overview of the risk of bias in the studies analyzed. (**b**) Detailed percentage ratings of bias risk for each category [3,8,9,10,11].

Figure 2 provides a summary of the risk of bias and the overall risk of bias score for each category in the articles reviewed. Figure 2a illustrates the risk of bias for each domain in every study, where green denotes a low risk of bias, yellow indicates an unclear risk, and red represents a high risk. The risk of bias in the included studies was assessed using five domains: (D1) the randomization process, (D2) deviations from intended interventions, (D3) missing outcome data, (D4) outcome measurement, and (D5) selection of the reported result. Most studies showed a low risk of bias across all domains. Rickert (2011) [11], Wildburger (2014) [9], Whitt (2020) [8], and Fatale (2022) [3] were judged to have low risk of bias in every domain. Fatale (2022) [3] exhibited a low risk in domain D1, leading to some concerns. Sauerbier (2011) [10] showed low risk in domains D1, D2, and D3 but raised some concerns in domains D4 and D5, resulting in an overall judgment of some concerns. The reasons regarding risk of bias are shown in Supplementary Materials Table S3. The overall risk of bias score for each category is presented as a percentage under the intention-to-treat principle in Figure 2b. The domains evaluated included the randomization process, deviations from intended interventions, missing outcome data, outcome measurement, and selection of the reported result. Most studies were deemed to have a low risk of bias (green) across key domains, with 100% of trials showing low risk for missing outcome data and deviations from intended interventions. The randomization process was mostly adequate, with 80% rated as low risk and the rest having some concerns (yellow). Some concerns were also noted in the measurement of outcomes and selection of reported results (20% each). Overall, while the majority of studies showed a low risk of bias, 40% were judged to have some concerns. No trial was rated as having a high risk of bias (red) in any domain.

### 3.3. Meta-Analysis

Five included articles (Fatale (2022) [3]; Whitt 2020 [8]; Wildburger (2014) [9] Sauerbier (2011) [10]; and Rickert (2011) [11]) assessed the efficacy of the additional use of stem cells in maxillary sinus augmentation.

#### 3.3.1. New Bone Formation

**Figure 3:**
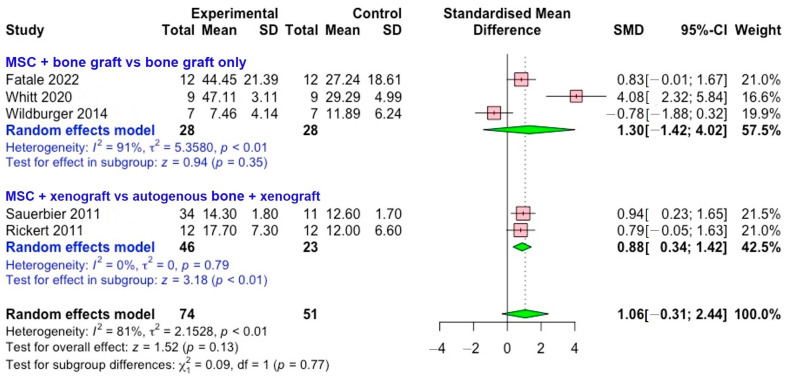
Forest plot illustrating the comparison between stem cell group compared with control for the new bone formation in sinus augmentation [3,8,9,10,11].

Figure 3 shows forest plot illustrating the effect of mesenchymal stem cells compared with different grafting materials on new bone formation. The analysis was conducted in two subgroups: MSCs combined with bone graft material versus bone graft only and MSC combined with xenograft versus autogenous bone combined xenograft. For the bone graft only subgroup, three studies (Fatale (2022) [3], Whitt (2020) [8], and Wildburger (2014) [9]) were used. This subgroup demonstrated considerable heterogeneity (*I*^2^ = 91%), suggesting substantial variability in study outcomes and potentially reflecting differences in study design, patient characteristics, or methodological approaches. The pooled SMD was 1.30 (95% CI of –1.42 to 4.02; *p* = 0.35), with significant heterogeneity observed (*I*^2^ = 91%).

In contrast, the autogenous bone subgroup (two studies: Sauerbier (2011) [10] and Rickert (2011) [11]) showed a pooled SMD of 0.88 (95% CI of 0.34 to 1.42; *p* < 0.01). These results suggest that MSCs combined with xenograft may provide more favorable results when compared with xenograft mixed with autogenous bone. Importantly, this subgroup showed no evidence of heterogeneity (*I*^2^ = 0%), strengthening the reliability and consistency of these findings.

When considering all five included studies together (total sample size: experimental n = 74, control n = 51), the overall effect demonstrated an SMD of 1.06 (95% CI of −0.31 to 2.44), with a test for overall effect yielding z = 1.52 (*p* = 0.13). This suggests a positive trend toward improved outcomes with MSC use, though the effect did not reach statistical significance at the pooled level. Heterogeneity across the combined dataset remained high (*I*^2^ = 81%), which indicates variability between studies. The test for subgroup differences was not significant (*χ*^2^ = 0.09, *p* = 0.77), implying that the apparent differences between MSC versus bone graft only and MSC versus autogenous bone were not statistically distinct.

#### 3.3.2. Publication Bias Analysis

**Figure 4:**
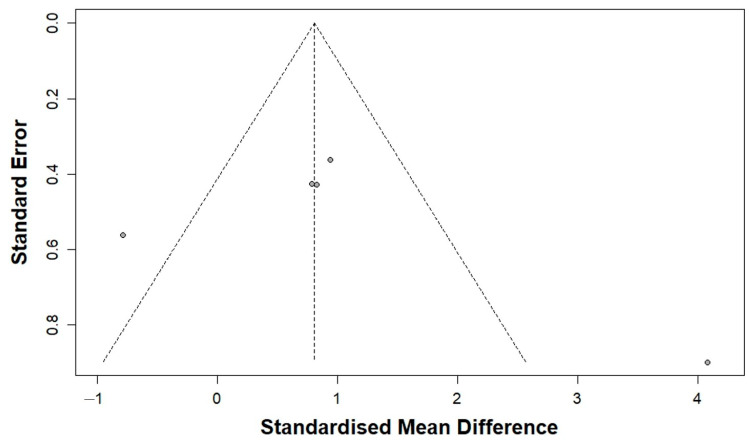
Funnel plot of publication bias analysis.

Table 2 presents the findings from the publication bias analysis. Visual inspection of the funnel plots suggested slight asymmetry (Figure 4). However, the trim-and-fill analysis did not identify any potentially missing studies, and the adjusted pooled effect size remained consistent with the original estimate. The initial meta-analysis indicated a significant mean difference (MD) of 0.94, with a 95% confidence interval ranging from 0.38 to 1.51 and a *p*-value of less than 0.01. The trim-and-fill method showed no need for imputation. Additionally, Egger’s regression test did not identify any significant small-study effects (*p* = 0.45), indicating an absence of publication bias.

### 3.4. Certainty of Evidence

According to the GRADE assessment, the certainty of evidence for new bone formation was downgraded to moderate. Although all included studies were randomized controlled trials, the presence of substantial heterogeneity and small sample sizes reduced confidence in the pooled results. The autogenous bone subgroup showed consistent findings and was rated as high certainty, whereas the bone graft–only subgroup was downgraded due to variability in outcomes. No publication bias was detected based on funnel plot symmetry and non-significant Egger’s test results. Overall, the evidence provides moderate confidence that stem cell–based grafting may improve bone regeneration in sinus augmentation.

## 4. Discussion

This systematic review and meta-analysis aimed to assess the effectiveness of MSCs in sinus floor augmentation by comparing new bone formation between groups with and without MSCs. The study’s results indicated a positive trend towards better outcomes with MSC use, although this effect did not achieve statistical significance at the pooled level. Additionally, MSCs combined with xenograft might yield more favorable results compared to autogenous bone combined with xenograft.

Sinus floor augmentation can be executed using either the crestal or lateral approach. In one study, five out of twelve patients underwent sinus augmentation via the crestal approach, while seven patients received it through the lateral approach [3]. For the crestal approach, osteotomes with smooth, gradually increasing diameters were used to expand the osteotomy until it was slightly smaller than the intended implant diameter, after which the sinus floor was carefully fractured and elevated using the osteotome [3]. In the lateral approach, a lateral window osteotomy was created in the lateral wall of the maxillary sinus using a round diamond bur [9,10], piezoelectric devices [3], or a combination of diamond bur and piezosurgical tools [8].

The choice of bone graft material is crucial for the success of sinus augmentation procedures, as it affects graft stability, new bone formation, and the long-term survival of implants [12]. Autogenous bone is often considered the gold standard due to its excellent osteoconductive, osteoinductive, and osteogenic properties [13]. However, its clinical application is limited by issues such as donor site morbidity and restricted availability [14]. Allografts have been widely used as an alternative to autogenous grafts [15]. Xenografts are frequently employed in sinus augmentation for their osteoconductive scaffolds, although they lack inherent osteogenic potential and may have slower resorption rates [16]. Alloplastic materials, like synthetic ceramics and bioactive composites, have shown positive results when used alone or with biological enhancers, such as platelet concentrates or mesenchymal stem cells, to further enhance bone regeneration [17]. In this meta-analysis, a synthetic bone graft made of β-tricalcium phosphate (80%) and hydroxyapatite (20%) was used, demonstrating biocompatibility and a resorption profile similar to autologous bone [3]. The studies in this meta-analysis used different types of graft materials. Mesenchymal stem cells were combined with allografts [8], xenografts [9,10,18], or synthetic bone substitutes [3]. The findings from this meta-analysis indicated that MSCs combined with xenografts might yield better results compared to xenografts mixed with autogenous bone. Overall, these results suggest that while autogenous bone is often the first choice, alternative grafting materials, including MSCs, can achieve predictable and successful outcomes in sinus augmentation when selected appropriately.

The source of mesenchymal stem cells can impact the results of sinus augmentation [19]. In the studies included, stem cells were obtained either from the periosteum [3] or from iliac crest bone marrow [9,10,18]. In one study, the periosteum was harvested after the primary flap was elevated [3]. Periosteum-derived cells are more easily accessible and have a strong proliferative capacity [20], while bone marrow-derived stem cells are known for their osteogenic differentiation potential [21]. These biological differences may lead to variability in new bone formation and implant integration across studies. The number of cells varied among studies, and the quantity of stem cells is a critical factor [22]. In the studies included in this meta-analysis, the sources and amounts reported were as follows: periosteum (5 × 5 mm) [3], bone marrow (52–60 mL) [9,10], and a commercially available product (250,000 cells/cm^3^, 2 g) [17]. Healing periods also varied, including 90 days [3], 14 weeks [8], 3–4 months [10], 3 and 6 months [9], and 12 months [18]. To minimize heterogeneity and allow for a more consistent comparison, this analysis used the 3-month follow-up data from Wildburger (2014) [9].

Even with the thorough methodology employed in this meta-analysis, which involved an extensive search strategy and strict compliance with PRISMA guidelines, several limitations must be recognized [23]. Firstly, due to the nature of the interventions, which involved either obtaining mesenchymal stem cells from an additional surgical site or using commercially available products [24], blinding of participants was likely impractical. Secondly, the statistical analysis indicated significant heterogeneity among the included studies [25], although no such heterogeneity was found within the autogenous bone subgroup. Additionally, cell-based approaches in the included studies should be interpreted cautiously, as the cellular components were not uniformly defined or verified. Lastly, differences in follow-up periods and patient demographics may restrict the applicability of the findings in clinical settings and should be taken into account [26]. Future research should focus on the use of standardized, well-characterized stem cell preparations and controlled study designs to accurately assess their true effect on bone regeneration.

## 5. Conclusions

The study’s results showed a positive trend towards improved outcomes with the use of MSCs, although this effect did not achieve statistical significance at the pooled level. Additionally, MSCs combined with xenograft might yield better results compared to autogenous bone combined with xenograft. These findings indicate potential clinical advantages, necessitating further standardized research to refine protocols and verify long-term outcomes.

## Figures and Tables

**Table 1:** Main characteristics of the included studies.

Study Author (Year)	Study Design	Sample Size (Test:Control) (Sites)	Test Procedure	Control Procedure	Results	Follow-Up
Fatale (2022) [3]	Controlled clinical trial	12:12	Mesenchymal stem cells (periosteal progenitor cells) + synthetic resorbable biphasic calcium phosphate	Synthetic resorbable biphasic calcium phosphate	Patients who received grafts with MSCs experienced a 63.18% increase in bone formation compared to those who received grafts without MSCs.	3 months
Whitt (2020) [8]	Single-center randomized controlled trial	9:9	Stem cell-based allograft (Osteocel Plus; NuVasive Therapeutics, San Diego, CA, USA),	Cortico-cancellous allograft	The findings indicated a statistically significant variation in the percentage of vital bone between the test and control groups at the posterior grafted locations.	3 months
Wildburger (2014) [9]	Split-mouth study	7:7	Mesenchymal stem cells (autogenous concentrated bone marrow aspirate) + pure bovine bone material	Pure bovine bone material	In the control group, new bone formation was recorded at 11.8% (SD 6.2%) after three months, while the test group exhibited 7.4% (SD 4.1%). At the six-month mark, the control group had 13.9% (SD 8.5%) new bone growth, compared to 13.5% (SD 5.4%) in the test group.	3 months, 6 months
Sauerbier (2011) [10]	Multicentric, randomized, controlled, clinical trial	11:34	Mesenchymal stem cells (bone marrow aspirate concentrate) + bovine bone mineral	30% autogenous bone + 70% bovine bone mineral	After a period of 3–4 months, the formation of new bone in the sinus is comparable when enhanced with either bone marrow aspirate concentrate and bovine bone mineral or a combination of autogenous bone and bovine bone mineral.	3–4 months
Rickert (2011) [11]	Randomized, controlled, split-mouth design study	12:12	Mesenchymal stem cells (iliac crest bone marrow concentrate) + bovine bone material	30% autogenous bone + 70% bovine bone material	Radiographic examination analysis revealed a marginal bone loss of 0.47 ± 0.31 mm on the test side and 0.41 ± 0.25 mm on the control side.	12 months

MSC, mesenchymal stem cell; SD, standardized deviation.

**Table 2:** Analyses for publication bias.

	Original Analysis		Trim-and-Fill Analysis		Egger’s Regression Test *p*-Value
	SMD (95% CI)	*p*-Value	SMD (95% CI)	Trimmed Studies/Total Studies	
**New bone formation**	0.94 (0.38 to 1.51)	*p* < 0.01	0.94 (0.38 to 1.51)	0/6	0.45

SMD, standardized mean difference; CI, confidence interval.

## Data Availability

The original contributions presented in the study are included in the article/Supplementary Materials, further inquiries can be directed to the corresponding authors.

## Supplementary Materials

The following supporting information can be downloaded at: https://www.mdpi.com/article/10.3390/bioengineering12111209/s1, Table S1: Search strategy of the online databases; Table S2: Excluded studies from full-text reading; Table S3: Risk of bias. References [5,18,27,28,29,30,31,32,33,34,35,36,37,38,39,40,41,42,43,44] are cited in the Supplementary Materials.

## Abbreviations

The following abbreviations are used in this manuscript:

SMD	standardized mean difference
CI	confidence interval
