# Supplementary material for: Clinical Application of Cell-Based Approaches in Maxillary Sinus Floor Augmentation: A Systematic Review and Meta-Analysis
Source: Bioengineering (Basel). 2025 Nov 5;12(11):1209. doi: 10.3390/bioengineering12111209 (PMC12650367; doi:10.3390/bioengineering12111209)
Supplement: Supplementary file 1 [file bioengineering-12-01209-s001.zip › bioengineering-3936402-supplementary.pdf]

Supplementary Table S1: Search strategy of the online databases.

DB	Search	Search strategy
**Medline via PubMed**	#1	"Sinus Floor Augmentation"[Mesh]
	#2	"Sinus Floor Augmentation"[TW] OR "Augmentation, Sinus Floor"[TW] OR "Augmentations, Sinus Floor"[TW] OR "Floor Augmentation, Sinus"[TW] OR "Floor Augmentations, Sinus"[TW] OR "Sinus Floor Augmentations"[TW] OR "Maxillary Sinus Floor Augmentation"[TW] OR "Sinus Augmentation Therapy"[TW] OR "Augmentation Therapies, Sinus"[TW] OR "Augmentation Therapy, Sinus"[TW] OR "Sinus Augmentation Therapies"[TW] OR "Therapies, Sinus Augmentation"[TW] OR "Therapy, Sinus Augmentation"[TW] OR "maxillary sinus augmentation"[TW] OR "maxillary sinus augmentations"[TW] OR "Sinus Augmentation"[TW] OR "Maxillary Augmentation"[TW] OR "maxillary sinus floor elevation"[TW]
	**#3 Combine**	**#1 OR #2**
	#4	"Stem Cells"[Mesh]
	#5	"Stem Cells"[TW] OR "Cells, Stem"[TW] OR "Cell, Stem"[TW] OR "Stem Cell"[TW] OR "Mother Cells"[TW] OR "Cell, Mother"[TW] OR "Cells, Mother"[TW] OR "Mother Cell"[TW] OR "Progenitor Cells"[TW] OR "Cell, Progenitor"[TW] OR "Cells, Progenitor"[TW] OR "Progenitor Cell"[TW] OR "Colony-Forming Unit"[TW] OR "Colony Forming Unit"[TW] OR "Colony-Forming Units"[TW] OR "Colony Forming Units"[TW]
	#6	"Mesenchymal Stem Cells"[Mesh]
	#7	"Mesenchymal Stem Cells"[TW] OR "Stem Cell, Mesenchymal"[TW] OR "Mesenchymal Stem Cell"[TW] OR "Stem Cells, Mesenchymal"[TW] OR "Mesenchymal Stromal Cells"[TW] OR "Mesenchymal Stromal Cell"[TW] OR "Stromal Cell, Mesenchymal"[TW] OR "Stromal Cells, Mesenchymal"[TW] OR "Wharton Jelly Cells"[TW] OR "Wharton's Jelly Cells"[TW] OR "Wharton's Jelly Cell"[TW] OR "Whartons Jelly Cells"[TW] OR "Bone Marrow Stromal Cells"[TW] OR "Bone Marrow Stromal Cell"[TW] OR "Bone Marrow Stromal Cells, Multipotent"[TW] OR "Multipotent Bone Marrow Stromal Cell"[TW] OR "Multipotent Bone Marrow Stromal Cells"[TW] OR "Bone Marrow Stromal Stem Cells"[TW] OR "Mesenchymal Progenitor Cell"[TW] OR "Mesenchymal Progenitor Cells"[TW] OR "Progenitor Cell, Mesenchymal"[TW] OR "Progenitor Cells, Mesenchymal"[TW] OR "Multipotent Mesenchymal Stromal Cells"[TW] OR "Mesenchymal Stromal Cells, Multipotent"[TW] OR "Multipotent Mesenchymal Stromal Cell"[TW] OR "Bone Marrow Mesenchymal Stem Cells"[TW] OR "Bone Marrow Mesenchymal Stem Cell"[TW] OR "Adipose-Derived Mesenchymal Stem Cells"[TW] OR "Adipose Derived Mesenchymal Stem Cells"[TW] OR "Adipose-Derived Mesenchymal Stromal Cells"[TW] OR "Adipose Derived Mesenchymal Stromal Cells"[TW] OR "Mesenchymal Stem Cells, Adipose-Derived"[TW] OR "Mesenchymal Stem Cells, Adipose Derived"[TW] OR "Adipose Tissue-Derived Mesenchymal Stromal Cell"[TW] OR "Adipose Tissue Derived Mesenchymal Stromal Cell"[TW] OR "Adipose Tissue-Derived Mesenchymal Stromal Cells"[TW] OR "Adipose Tissue Derived Mesenchymal Stromal Cells"[TW] OR "Adipose Tissue-Derived Mesenchymal Stem Cell"[TW] OR "Adipose Tissue Derived Mesenchymal Stem Cell"[TW] OR "Adipose Tissue-Derived Mesenchymal Stem Cells"[TW] OR "Adipose Tissue Derived Mesenchy-

		mal Stem Cells"[TW] OR "Adipose-Derived Mesenchymal Stem Cell"[TW] OR "Adipose Derived Mesenchymal Stem Cell"[TW]
	**#8 Combine**	**#4 OR #5 OR #6 OR #7**
	**#9 Combine**	**#3 AND #8**
	**#10 Limit**	**#9 AND (alladult[Filter])**
	**#11 Limit**	**#10 NOT ("animals"[MeSH] NOT "Humans"[MeSH])**
**Embase**	#1	"sinus floor augmentation"/exp
	#2	"Sinus Floor Augmentation":ti,ab,kw,de OR "Augmentation, Sinus Floor":ti,ab,kw,de OR "Augmentations, Sinus Floor":ti,ab,kw,de OR "Floor Augmentation, Sinus":ti,ab,kw,de OR "Floor Augmentations, Sinus":ti,ab,kw,de OR "Sinus Floor Augmentations":ti,ab,kw,de OR "Maxillary Sinus Floor Augmentation":ti,ab,kw,de OR "Sinus Augmentation Therapy":ti,ab,kw,de OR "Augmentation Therapies, Sinus":ti,ab,kw,de OR "Augmentation Therapy, Sinus":ti,ab,kw,de OR "Sinus Augmentation Therapies":ti,ab,kw,de OR "Therapies, Sinus Augmentation":ti,ab,kw,de OR "Therapy, Sinus Augmentation":ti,ab,kw,de OR "maxillary sinus augmentation":ti,ab,kw,de OR "maxillary sinus augmentations":ti,ab,kw,de OR "Sinus Augmentation":ti,ab,kw,de OR "Maxillary Augmentation":ti,ab,kw,de OR "maxillary sinus floor elevation":ti,ab,kw,de
	**#3 Combine**	**#1 OR #2**
	#4	"stem cell"/exp
	#5	"Stem Cells":ti,ab,kw,de OR "Cells, Stem":ti,ab,kw,de OR "Cell, Stem":ti,ab,kw,de OR "Stem Cell":ti,ab,kw,de OR "Mother Cells":ti,ab,kw,de OR "Cell, Mother":ti,ab,kw,de OR "Cells, Mother":ti,ab,kw,de OR "Mother Cell":ti,ab,kw,de OR "Progenitor Cells":ti,ab,kw,de OR "Cell, Progenitor":ti,ab,kw,de OR "Cells, Progenitor":ti,ab,kw,de OR "Progenitor Cell":ti,ab,kw,de OR "Colony-Forming Unit":ti,ab,kw,de OR "Colony Forming Unit":ti,ab,kw,de OR "Colony-Forming Units":ti,ab,kw,de OR "Colony Forming Units":ti,ab,kw,de
	#6	"mesenchymal stem cell"/exp
	#7	"Mesenchymal Stem Cells":ti,ab,kw,de OR "Stem Cell, Mesenchymal":ti,ab,kw,de OR "Mesenchymal Stem Cell":ti,ab,kw,de OR "Stem Cells, Mesenchymal":ti,ab,kw,de OR "Mesenchymal Stromal Cells":ti,ab,kw,de OR "Mesenchymal Stromal Cell":ti,ab,kw,de OR "Stromal Cell, Mesenchymal":ti,ab,kw,de OR "Stromal Cells, Mesenchymal":ti,ab,kw,de OR "Wharton Jelly Cells":ti,ab,kw,de OR "Wharton^s Jelly Cells":ti,ab,kw,de OR "Wharton^s Jelly Cell":ti,ab,kw,de OR "Whartons Jelly Cells":ti,ab,kw,de OR "Bone Marrow Stromal Cells":ti,ab,kw,de OR "Bone Marrow Stromal Cell":ti,ab,kw,de OR "Bone Marrow Stromal Cells, Multipotent":ti,ab,kw,de OR "Multipotent Bone Marrow Stromal Cell":ti,ab,kw,de OR "Multipotent Bone Marrow Stromal Cells":ti,ab,kw,de OR "Bone Marrow Stromal Stem Cells":ti,ab,kw,de OR "Mesenchymal Progenitor Cell":ti,ab,kw,de OR "Mesenchymal Progenitor Cells":ti,ab,kw,de OR "Progenitor

		Cell, Mesenchymal":ti,ab,kw,de OR "Progenitor Cells, Mesenchymal":ti,ab,kw,de OR "Multipotent Mesenchymal Stromal Cells":ti,ab,kw,de OR "Mesenchymal Stromal Cells, Multipotent":ti,ab,kw,de OR "Multipotent Mesenchymal Stromal Cell":ti,ab,kw,de OR "Bone Marrow Mesenchymal Stem Cells":ti,ab,kw,de OR "Bone Marrow Mesenchymal Stem Cell":ti,ab,kw,de OR "Adipose-Derived Mesenchymal Stem Cells":ti,ab,kw,de OR "Adipose Derived Mesenchymal Stem Cells":ti,ab,kw,de OR "Adipose-Derived Mesenchymal Stromal Cells":ti,ab,kw,de OR "Adipose Derived Mesenchymal Stromal Cells":ti,ab,kw,de OR "Mesenchymal Stem Cells, Adipose-Derived":ti,ab,kw,de OR "Mesenchymal Stem Cells, Adipose Derived":ti,ab,kw,de OR "Adipose Tissue-Derived Mesenchymal Stromal Cell":ti,ab,kw,de OR "Adipose Tissue Derived Mesenchymal Stromal Cell":ti,ab,kw,de OR "Adipose Tissue-Derived Mesenchymal Stromal Cells":ti,ab,kw,de OR "Adipose Tissue Derived Mesenchymal Stromal Cells":ti,ab,kw,de OR "Adipose Tissue-Derived Mesenchymal Stem Cell":ti,ab,kw,de OR "Adipose Tissue Derived Mesenchymal Stem Cell":ti,ab,kw,de OR "Adipose Tissue-Derived Mesenchymal Stem Cells":ti,ab,kw,de OR "Adipose Tissue Derived Mesenchymal Stem Cells":ti,ab,kw,de OR "Adipose-Derived Mesenchymal Stem Cell":ti,ab,kw,de OR "Adipose Derived Mesenchymal Stem Cell":ti,ab,kw,de
	**#8 Combine**	**#4 OR #5 OR #6 OR #7**
	**#9 Combine**	**#3 AND #8**
	**#10 Limit**	**#9 AND ([adult]/lim OR [young adult]/lim OR [middle aged]/lim OR [aged]/lim OR [very elderly]/lim)**
	**#11 Limit**	**#10 NOT ('animal'/exp NOT 'human'/exp)**
**Cochrane Library**	#1	[mh "Sinus Floor Augmentation"]
	#2	"Sinus Floor Augmentation":ti,ab,kw OR "Augmentation, Sinus Floor":ti,ab,kw OR "Augmentations, Sinus Floor":ti,ab,kw OR "Floor Augmentation, Sinus":ti,ab,kw OR "Floor Augmentations, Sinus":ti,ab,kw OR "Sinus Floor Augmentations":ti,ab,kw OR "Maxillary Sinus Floor Augmentation":ti,ab,kw OR "Sinus Augmentation Therapy":ti,ab,kw OR "Augmentation Therapies, Sinus":ti,ab,kw OR "Augmentation Therapy, Sinus":ti,ab,kw OR "Sinus Augmentation Therapies":ti,ab,kw OR "Therapies, Sinus Augmentation":ti,ab,kw OR "Therapy, Sinus Augmentation":ti,ab,kw OR "maxillary sinus augmentation":ti,ab,kw OR "maxillary sinus augmentations":ti,ab,kw OR "Sinus Augmentation":ti,ab,kw OR "Maxillary Augmentation":ti,ab,kw OR "maxillary sinus floor elevation":ti,ab,kw
	**#3 Combine**	**#1 OR #2**
	#4	[mh "Stem Cells"]
	#5	"Stem Cells":ti,ab,kw OR "Cells, Stem":ti,ab,kw OR "Cell, Stem":ti,ab,kw OR "Stem Cell":ti,ab,kw OR "Mother Cells":ti,ab,kw OR "Cell, Mother":ti,ab,kw OR "Cells, Mother":ti,ab,kw OR "Mother Cell":ti,ab,kw OR "Progenitor Cells":ti,ab,kw OR "Cell, Progenitor":ti,ab,kw OR "Cells, Progenitor":ti,ab,kw OR "Progenitor Cell":ti,ab,kw OR "Colony-Forming Unit":ti,ab,kw OR "Colony Forming Unit":ti,ab,kw OR "Colony-Forming

	Units":ti,ab,kw OR "Colony Forming Units":ti,ab,kw
#6	[mh "Mesenchymal Stem Cells"]
#7	"Mesenchymal Stem Cells":ti,ab,kw OR "Stem Cell, Mesenchymal":ti,ab,kw OR "Mesenchymal Stem Cell":ti,ab,kw OR "Stem Cells, Mesenchymal":ti,ab,kw OR "Mesenchymal Stromal Cells":ti,ab,kw OR "Mesenchymal Stromal Cell":ti,ab,kw OR "Stromal Cell, Mesenchymal":ti,ab,kw OR "Stromal Cells, Mesenchymal":ti,ab,kw OR "Wharton Jelly Cells":ti,ab,kw OR "Wharton's Jelly Cells":ti,ab,kw OR "Wharton's Jelly Cell":ti,ab,kw OR "Whartons Jelly Cells":ti,ab,kw OR "Bone Marrow Stromal Cells":ti,ab,kw OR "Bone Marrow Stromal Cell":ti,ab,kw OR "Bone Marrow Stromal Cells, Multipotent":ti,ab,kw OR "Multipotent Bone Marrow Stromal Cell":ti,ab,kw OR "Multipotent Bone Marrow Stromal Cells":ti,ab,kw OR "Bone Marrow Stromal Stem Cells":ti,ab,kw OR "Mesenchymal Progenitor Cell":ti,ab,kw OR "Mesenchymal Progenitor Cells":ti,ab,kw OR "Progenitor Cell, Mesenchymal":ti,ab,kw OR "Progenitor Cells, Mesenchymal":ti,ab,kw OR "Multipotent Mesenchymal Stromal Cells":ti,ab,kw OR "Mesenchymal Stromal Cells, Multipotent":ti,ab,kw OR "Multipotent Mesenchymal Stromal Cell":ti,ab,kw OR "Bone Marrow Mesenchymal Stem Cells":ti,ab,kw OR "Bone Marrow Mesenchymal Stem Cell":ti,ab,kw OR "Adipose-Derived Mesenchymal Stem Cells":ti,ab,kw OR "Adipose Derived Mesenchymal Stem Cells":ti,ab,kw OR "Adipose-Derived Mesenchymal Stromal Cells":ti,ab,kw OR "Adipose Derived Mesenchymal Stromal Cells":ti,ab,kw OR "Mesenchymal Stem Cells, Adipose-Derived":ti,ab,kw OR "Mesenchymal Stem Cells, Adipose Derived":ti,ab,kw OR "Adipose Tissue-Derived Mesenchymal Stromal Cell":ti,ab,kw OR "Adipose Tissue Derived Mesenchymal Stromal Cell":ti,ab,kw OR "Adipose Tissue-Derived Mesenchymal Stromal Cells":ti,ab,kw OR "Adipose Tissue Derived Mesenchymal Stromal Cells":ti,ab,kw OR "Adipose Tissue-Derived Mesenchymal Stem Cell":ti,ab,kw OR "Adipose Tissue Derived Mesenchymal Stem Cell":ti,ab,kw OR "Adipose Tissue-Derived Mesenchymal Stem Cells":ti,ab,kw OR "Adipose Tissue Derived Mesenchymal Stem Cells":ti,ab,kw OR "Adipose-Derived Mesenchymal Stem Cell":ti,ab,kw OR "Adipose Derived Mesenchymal Stem Cell":ti,ab,kw
**#8 Combine**	**#4 OR #5 OR #6 OR #7**
**#9 Combine**	**#3 AND #8**
**#10 Limit**	**#9 AND [mh "Adult"]**
**#11 Limit**	**#9 NOT ([mh "animals"] NOT [mh "Humans"])**
**#6 Combine**	**#4 OR #5**
**#7 Combine**	**#3 AND #6**

Supplementary Table S2: Excluded studies from full-text reading.

Reasons for exclusion	References
**Comment/ Letter**	1. Tzur, E.; Rozen, N.; David, D.B.; Barzilai, M.G.; Novak, A.; Kivity, V.; Bronshtein, T.; Meretzki, S. Advancing mesenchymal cell–based bone tissue engineering: Comprehensive phase II clinical trial outcomes of BonoFill in maxillofacial reconstruction. *Cytotherapy* **2024**, *26*, S20-S21, doi:10.1016/j.jcyt.2024.03.493. [27]<br>2. Tzur, E.; Bronshtein, T. Complicated large maxillofacial bone deficiencies successfully reconstructed using BonoFill: A novel tissue-engineered bone graft derived from patient-specific adipose tissue–based osteoprogenitor cells. *Int J Oral Maxillofac Surg* **2024**, *52*, 12-13, doi:10.1016/j.ijom.2023.10.044. [28]<br>3. Nulend, J.K.; Guasch, E.F.; Bravenboer, N.; Helder, M.N.; Bruggenkate, C.M.T.; Schulten, E.A.J.M. Blood vessel formation and bone regeneration potential of human adipose stem cells for jaw bone augmentation. *BioImpacts* **2018**, *8*, 25, doi:10.15171/bi.2018.S1. [29]<br>4. Forouzanfar, T.; Nulend, J.K.; Prins, H.J.; Schulten, E.A.J.M.; Bruggenkate, C.M.T.; Helder, M.N. Clinical implementation of the one-step surgical procedure for craniofacial dental implantation in the maxillary sinus floor elevation (MSFE) model. *BioImpacts* **2018**, *8*, 6, doi:10.15171/bi.2018.S1. [30]<br>5. Schulten, E.A.J.M.; Prins, H.J.; Ten Bruggenkate, C.M.; Klein Nulend, J.; Helder, M.N. Bone regeneration with adipose stem cells and calcium phosphate ceramics in the human maxillary sinus floor elevation model using a one-step surgical procedure. *Int J Oral Maxillofac Surg* **2017**, *46*, 214, doi:10.1016/j.ijom.2017.02.726. [31]
**Animal study**	1. Jensen, T. Radiographic outcomes following maxillary sinus floor augmentation with allogeneic adipose tissue–derived stem cells seeded on deproteinized bovine bone mineral: A randomized controlled trial in minipigs. *Int J Oral Maxillofac Surg* **2024**, *52*, 15, doi:10.1016/j.ijom.2023.10.050. [32]<br>2. Starch-Jensen, T.; Aludden, H.; Dahlin, C.; Bruun, N.H.; Fink, T. Histomorphometric outcome following sinus floor augmentation with allogeneic adipose tissue-derived stem cells. A randomized controlled experimental study. *J. Craniomaxillofac Surg* **2024**, doi:10.1016/j.jcms.2024.11.008. [5]
**Duplicate**	1. Prins, H.J.; Schulten, E.A.J.M.; Ten Bruggenkate, C.M.; Klein-Nulend, J.; Helder, M.N. Bone regeneration using the freshly isolated autologous stromal vascular fraction of adipose tissue in combination with calcium phosphate ceramics. *Stem Cells Transl Med* **2016**, *5*, 1362-1374, doi:10.5966/sctm.2015-0369. [33]<br>2. Shayesteh, Y.S.; Khojasteh, A.; Soleimani, M.; Alikhasi, M.; Khoshzaban, A.; Ahmadbeigi, N. Sinus augmentation using human mesenchymal stem cells loaded into a β-tricalcium phosphate/hydroxyapatite scaffold. *Oral Surg Oral Med Oral Pathol Oral Radiol Endod* **2008**, *106*, 203-209, doi:10.1016/j.tripleo.2007.12.001. [34]

**No randomized controlled trial**	1. Tzur, E.; Ben-David, D.; Gur Barzilai, M.; Rozen, N.; Meretzki, S. Safety and efficacy results of BonoFill first-in-human, phase I/IIa clinical trial for the maxillofacial indication of sinus augmentation and mandibular bone void filling. *J Oral Maxillofac Surg* **2021**, *79*, 787-798.e782, doi:10.1016/j.joms.2020.12.010. [35]<br>2. Farré-Guasch, E.; Bravenboer, N.; Helder, M.N.; Schulten, E.; Ten Bruggenkate, C.M.; Klein-Nulend, J. Blood vessel formation and bone regeneration potential of the stromal vascular fraction seeded on a calcium phosphate scaffold in the human maxillary sinus floor elevation model. *Materials (Basel)* **2018**, *11*, doi:10.3390/ma11010161. [36]<br>3. Katagiri, W.; Watanabe, J.; Toyama, N.; Osugi, M.; Sakaguchi, K.; Hibi, H. Clinical study of bone regeneration by conditioned medium from mesenchymal stem cells after maxillary sinus floor elevation. *Implant Dent* **2017**, *26*, 607-612, doi:10.1097/id.0000000000000618. [37]<br>4. Prins, H.J.; Schulten, E.A.; Ten Bruggenkate, C.M.; Klein-Nulend, J.; Helder, M.N. Bone regeneration using the freshly isolated autologous stromal vascular fraction of adipose tissue in combination with calcium phosphate ceramics. *Stem Cells Transl Med* **2016**, *5*, 1362-1374, doi:10.5966/sctm.2015-0369. [33]<br>5. Duttenhoefer, F.; Hieber, S.F.; Stricker, A.; Schmelzeisen, R.; Gutwald, R.; Sauerbier, S. Follow-up of implant survival comparing ficoll and bone marrow aspirate concentrate methods for hard tissue regeneration with mesenchymal stem cells in humans. *BioResearch Open Access* **2014**, *3*, 75-76, doi:10.1089/biores.2014.0003. [39]<br>6. Yamada, Y.; Nakamura, S.; Ueda, M.; Ito, K. Osteotome technique with injectable tissue-engineered bone and simultaneous implant placement by cell therapy. *Clin Oral Implants Res* **2013**, *24*, 468-474, doi:10.1111/j.1600-0501.2011.02353.x. [40]<br>7. Gonshor, A.; McAllister, B.S.; Wallace, S.S.; Prasad, H. Histologic and histomorphometric evaluation of an allograft stem cell-based matrix sinus augmentation procedure. *Int J Oral Maxillofac Implants* **2011**, *26*, 123-131. [41]<br>8. Sauerbier, S.; Stricker, A.; Kuschnierz, J.; Bühler, F.; Oshima, T.; Xavier, S.P.; Schmelzeisen, R.; Gutwald, R. In vivo comparison of hard tissue regeneration with human mesenchymal stem cells processed with either the FICOLL method or the BMAC method. *Tissue Eng Part C Methods* **2010**, *16*, 215-223, doi:10.1089/ten.TEC.2009.0269. [42]<br>9. Shayesteh, Y.S.; Khojasteh, A.; Soleimani, M.; Alikhasi, M.; Khoshzaban, A.; Ahmadbeigi, N. Sinus augmentation using human mesenchymal stem cells loaded into a beta-tricalcium phosphate/hydroxyapatite scaffold. *Oral Surg Oral Med Oral Pathol Oral Radiol Endod* **2008**, *106*, 203-209, doi:10.1016/j.tripleo.2007.12.001. [34]
**No relevant outcome**	1. Wu, V.; Klein-Nulend, J.; Bravenboer, N.; Ten Bruggenkate, C.M.; Helder, M.N.; Schulten, E. Long-term safety of bone regeneration using autologous stromal vascular fraction and calcium phosphate ceramics: A 10-year prospective cohort study. *Stem Cells Transl Med* **2023**, *12*, 617-630, doi:10.1093/stcltm/szad045. [44]<br>2. Gupta, A.S.; Aurora, J.K.; Dubey, K.N.; Chauhan, H.; Saxena, M.; Ganvir, S.R. A comparative evaluation of bone regeneration using mesenchymal stem cells versus blood coagulum in sinus augmentation procedures. *Natl J Maxillofac Surg* **2021**, *12*, 349-356, doi:10.4103/njms.njms_358_21. [45]<br>3. Bertolai, R.; Catelani, C.; Aversa, A.; Rossi, A.; Giannini, D.; Bani, D. Bone graft and mesenchymal stem cells: clinical observations and histological analysis. *Clin Cases Miner Bone Metab* **2015**, *12*, 183-187, doi:10.11138/ccmbm/2015.12.2.183. [46]

4. Rickert, D.; Vissink, A.; Slot, W.J.; Sauerbier, S.; Meijer, H.J.A.; Raghoebar, G.M. Maxillary sinus floor elevation surgery with BioOss® mixed with a bone marrow concentrate or autogenous bone: Test of principle on implant survival and clinical performance. *Int J Oral Maxillofac Surg* **2014**, *43*, 243-247, doi:10.1016/j.ijom.2013.09.006. [11]

Supplementary Table S3: Risk of bias.

Study	D1: Randomization process	D2: Deviations from the intended interventions	D3: Missing outcome data	D4: Measurement of the outcome	D5: Selection of the reported result
Fatale 2022	Some concerns : “Considering the small sample of the study, the assignment of patients to various groups was performed according to the dynamic criterion of minimization for the factor of age.”	Low risk: No deviation from the intended intervention has been reported.	Low risk: No dropouts were reported.	Low risk: “Blinded histomorphometric analysis was performed by an independent laboratory that was not a part of the research Group.”	Low risk: “Blinded histomorphometric analysis was performed by an independent laboratory that was not a part of the research group.”
Whitt 2020	Low risk: “Eligible patients were randomly assigned to one of two treatment groups using a randomization table generated by a computer.”	Low risk: No deviation From the Intended intervention has been reported.	Low risk: No dropouts were reported.	Low risk: “This study was a single-center, prospective, randomized, controlled clinical, and blinded histomorphometric study.”	Low risk: This study was a single-center, prospective, randomized, controlled clinical, and blinded histomorphometric study.”
Wildburger 2014	Low risk: “Preoperative randomizing was performed to determine test and control site in each case (Randomizer for Clinical Trials, Institute for Medical Informatics, Statistics and Documentation, Medical Univ. Graz).”	Low risk: No deviation From the Intended intervention has been reported.	Low risk: Nearly all data for outcome were available.	Low risk: “During the whole analysis, histologists were blinded to the samples’ groups.”	Low risk: “During the whole analysis, histologists were blinded to the samples’ groups.”

Sauerbier 2011	Low risk: “Each sinus was randomly assigned to either control or test arm. Randomization envelopes in blocks of six were generated in a 1:2 ratio for test and control.”	Low risk: No deviation from the intended intervention has been reported.	Low risk: “14 patients (25 sinus) were excluded because of protocol violations, which would have led to NBF bias. The main reason was that the implantation was outside the 3–4 months healing time”	Some concerns : Objective and standardized methods such as histomorphometry and CBCT volumetric measurements were used. However, the blinding status of the outcome assessors was not reported.	Some concerns: “This study is a pilot study, as no data could be identified to give a thorough base for a power analysis.”
Rickert 2011	Low risk: “Randomly, performed by envelopes, on one side the augmentation procedure was performed with bovine bone mineral seeded with MSCs harvested from the posterior iliac crest (test group) and BioOsss combined with autologous bone on the contralateral side (control group).”	Low risk: No deviation from the intended intervention has been reported.	Low risk: “All 12 patients were treated with a bilateral sinus floor augmentation procedure, but the results of the biopsies taken from one sinus (control side) of one patient were not included in this analysis as histological examination revealed that these biopsies were not taken from an augmented location.”	Low risk: “The histologists were blinded to the samples’ groups throughout the histomorphometrical analysis.”	Low risk: “The histologists were blinded to the samples’ groups throughout the histomorphometrical analysis.”
